# Two human MARs effectively increase transgene expression in transfected CHO cells

**DOI:** 10.1111/jcmm.14018

**Published:** 2018-11-18

**Authors:** Qin Li, Chun‐peng Zhao, Yan Lin, Chao Song, Fang Wang, Tian‐yun Wang

**Affiliations:** ^1^ Department of Biochemistry and Molecular Biology Xinxiang Medical University Henan China; ^2^ International Joint Research Laboratory for Recombiant Pharmaceutical Protein Expression System of Henan Xinxiang Medical University Xinxiang Henan China

**Keywords:** Chinese hamster ovaries cell, gene expression, matrix attachment region, transgene silencing

## Abstract

Matrix attachment regions (MARs) can enhance the expression level of transgene in Chinese hamster ovaries (CHO) cell expression system. However, improvements in function and analyses of the mechanism remains unclear. In this study, we screened two new and more functional MAR elements from the human genome DNA. The human MAR‐3 and MAR‐7 element were cloned and inserted downstream of the polyA site in a eukaryotic vector. The constructs were transfected into CHO cells, and screened under G418 to produce the stably transfected cell pools. The expression levels and stability of enhanced green fluorescent protein (eGFP) were detected by flow cytometry. The transgene copy number and transgene expression at mRNA level were detected by quantitative real‐time PCR. The results showed that the expression level of eGFP of cells transfected with MAR‐containing vectors were all higher than those of the vectors without MARs under transient and stably transfection. The enhancing effect of MAR‐7 was higher than that of MAR‐3. Additionally, we found that MAR significantly increased eGFP copy numbers and eGFP gene mRNA expression level as compared with the vector without. In conclusion, MAR‐3 and MAR‐7 gene can promote the expression of transgene in transfected CHO cells, and its effect may be related to the increase of the number of copies.

## 1 INTRODUCTION

Mammalian cell expression systems are generally the preferred platform for producing the recombinant therapy proteins, as they can generate the large and complex proteins with post‐translational modifications similar to those produced in humans.^1^, ^2^ More than 70% of the recombinant therapeutic proteins were produced by the Chinese hamster ovaries (CHO) cells.^3^ Low expression level and transgene silencing of recombinant protein in CHO cells are common problems in the production of recombinant protein.^4^, ^5^

Matrix attachment regions (MARs) are the most commonly used epigenetic regulatory element that enhance the integration of plasmids, participate in the formation of chromatin boundaries and enhance transcription. Therefore, MARs are widely used to improve the high level of expression of transgenic genes and to prevent epigenetic silencing by blocking the transmission of heterochromatin.^6^, ^7^ Up to now, it has been reported that several MAR elements can increase the level of transgene expression as well as the proportion of positive colonies in CHO cell expression systems, and also reduce the differences of expression between different transformed strains.^8^, ^9^, ^10^

Although it has been demonstrated that several MARs can increase the transgene expression in transfected CHO cells; however, improvements in function and analyses of the underlying mechanism are necessary. In this study, we identified two new, more powerful MAR elements from the human genome DNA, which can be used to improve the transgene expression in transfected CHO cells.

## 2 MATERIALS AND METHODS

### 2.1 Vector construction

A pIRES‐EGFP plasmid vector, as described in the previous study,^9^, ^10^, ^11^ was used in the present study. Human MAR‐3 (GenBank no. AC061708.17, position 61464‐62030) and MAR‐7 (GenBank no. X67858.1, position 2661‐3661) were artificially synthesized by General Biosystems (An'hui, China), and then cloned into the region downstream of the poly (A) sequence in the pIRES‐EGFP vector, thereby producing pIRES‐MAR‐3 and pIRES‐MAR‐7.

### 2.2 Cell culture and transfection

Chinese hamster ovaries cells (#A11557‐01; Life Technologies, Carlsbad, CA, USA) were cultured in Dulbecco's Modified Eagle's Medium+F12 (Gibco, Carlsbad, CA, USA) supplemented with 10% foetal bovine serum (FBS; Gibco) and 1% penicillin and streptomycin (Beyotime, Shanghai, China) at 37°C in a humidified incubator with 5% CO_2_. Cells at the exponential growth phase were collected and seeded into 24‐well plates at 2 × 10^6^ cells/well. The next day, the cells were divided into two groups for transfection with pIRES‐MAR‐3 and pIRES‐MAR‐7. pIRES‐EGFP was used as a control vector. Transfections were performed with Lipofectamine^®^ 2000 reagent (Invitrogen, Waltham, MA, USA) according to the manufacturer's instructions.

### 2.3 Screening of stably transfected CHO cells

About 48 hours after transfection, the cells were cultured in the presence of 800 μg/mL G418 (Invitrogen) for 2 weeks until the untransfected control cells had died. Subsequently, transfected cells were seeded (4 × 10^5^ cells/mL) in 6‐well plates under 500 μg/mL G418 selection pressure to produce the transfected cell pools for further analysis to determine fluorescence intensity.

### 2.4 Flow cytometry

eGFP expression was analysed by flow cytometry using 1 × 10^6^ CHO cells, with non‐transfected cells used as the negative control. The results were analysed with FlowJo software (Tree Star, Ashland, OR, USA). Three stably transfected pools were generated for each vector. eGFP expression in the cells was analysed with a FACS Calibur instrument (Becton Dickinson, Franklin Lakes, NJ, USA). A total of 100 000 fluorescent events were acquired using a 530/15 band‐pass filter for the green fluorescent protein signal acquired with a fluorescence emission wavelength of 530 nm.

### 2.5 Quantitative real‐time PCR analysis

Genomic DNA and total RNA were extracted according to the previous studies.^12^ Quantitative real‐time PCR (qRT‐PCR) was used to determine the eGFP mRNA levels, as well as the gene copy numbers. The glyceraldehyde phosphate dehydrogenase (GAPDH) gene was used as an internal reference. The sequence of primers designed with Primer 5 software are as follows: eGFP forward: 5′‐CTACGTCCAGGAGCGCACCATCT‐3′, reverse: 5′‐GTTCTTCTGCTTGTCGGCCATGATAT‐3′; GAPDH forward: 5′‐CGACCCCTTCATTGACCTC‐3′, GAPDH reverse: 5′‐CTCCACGACATACTCAGCACC‐3′. qRT‐PCRs were performed with the ABI 7500 SYBR Fluorescence quantitative PCR instrument (Applied Biosystems, Foster City, CA, USA), and 7500 Fast System SDS Software was used to analyse the results. The fold change in eGFP specific transcripts relative to the GAPDH endogenous control gene was determined by the 2^−∆∆Ct^ method.

### 2.6 Statistical analysis

All data were obtained from at least three independent experiments and were analysed using SPSS 18.0 software (SPSS Inc., Chicago, IL, USA). Data are reported as the mean ± SD. Group means were compared using single‐factor analysis of variance, and *t* tests were used for pairwise comparisons. Differences with *P* < 0.05 were considered statistically significant.

## 3 RESULTS

### 3.1 Transgene expression levels in stably transfected cells

**Figure 1:**
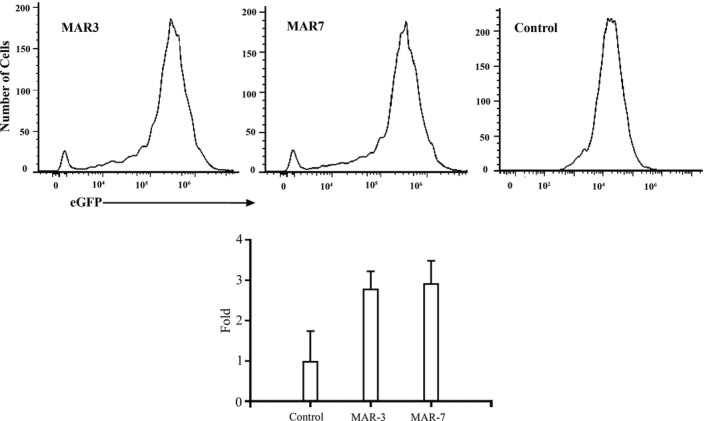
Analysis of eGFP MFI in stably transfected CHO cell line. (A) The eGFP MFI was measured after culturing cells 20 days under G418 pressure. Each value represents the average and standard deviation of three independent stably transfected pools. (B) Fold statistical analysis results of expression level, and the eGFP MFI were normalized to a control vector without MAR

The CHO cells were transfected by the vector containing MAR and without MAR, stably transfected clones were obtained under G418 screening pressure. The expression of eGFP in stably transfected cells was detected by flow cytometry (Figure 1A). The results showed that the mean fluorescence intensity (MFI) of eGFP containing MAR‐3 and MAR‐7 was 2.79‐ and 2.92‐fold compared with the control vector respectively (Figure 1B), suggesting two MAR elements can increase the transgene expression level of stably transfected cells.

### 3.2 qRT‐PCR analysis

**Figure 2:**
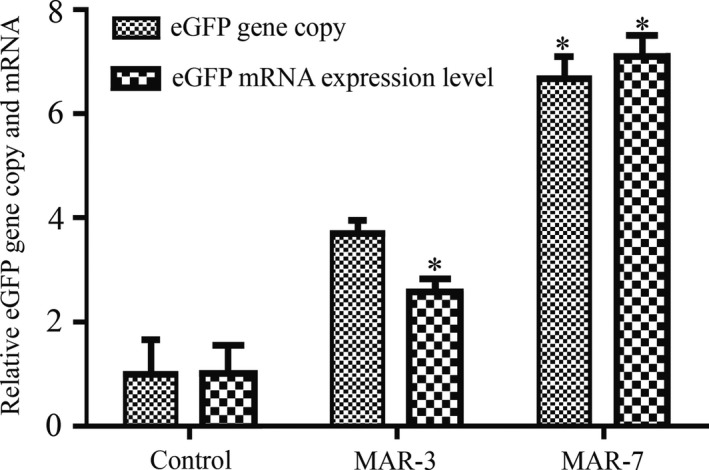
Analysis of the relative eGFP mRNA levels and eGFP copy number in stably transfected cell pools. Fluorescent quantitative PCR was used to measure relative eGFP mRNA levels and gene copy numbers. The 2^−∆∆Ct^ method was used to calculate relative values. The mRNA levels and eGFP gene copy numbers were normalized to the control whose value was set to 1. Three independent experiments were performed in this study. Standard error of the mean (SEM) is indicated (*P < 0.05)

To investigate the correlation between eGFP activity and its mRNA levels, we performed qRT‐PCR with cDNAs prepared from CHO cells transfected with MAR‐3 and MAR‐7 constructs. As shown in Figure 2, two MARs enhanced the accumulation of eGFP mRNA compared with that of the control plasmid. MAR‐3 and MAR‐7 can increase the eGFP expression by approximately 2.58‐ and 7.11‐fold respectively. This result suggested that eGFP activity correlates well with mRNA levels for the MAR‐containing vectors.

The relative copy number of eGFP gene transfected by MAR‐3 and MAR‐7 expression vectors was higher 3.73‐ and 6.70‐fold than that without MAR expression vector (Figure 2), suggesting that the increase in eGFP gene expression level is related to the increase in the number of copies.

## 4 DISCUSSION

In the present study, we investigated the effects of MAR‐3 and MAR‐7 on transgene expression in CHO cells using eGFP reporter gene. We found that the two MARs vectors could improve the transfection efficiency and the expression level of eGFP reporter gene and mAb compared with the control vector.

In the present study, MAR‐3 and MAR‐7 enhanced the transgene expression levels in stably transfected CHO cells, which are consistent with the previous studies.^8^, ^9^, ^10^ In addition, MAR‐7 showed the higher enhancing activity compared with MAR‐3. We suspect that the backbone, motif composition of MARs may determine their effect on transgene expression. According to a previous study, different MAR combination influence MAR activity with respect to increasing transgene expression.^8^, ^13^

In this study, we found that the increase in eGFP gene expression level is related to the increase in the number of copies of transgenic. It was also found that the activity of eGFP was closely related to the level of mRNA containing MAR expression vector.

In conclusion, in this study, we first identified two effective MAR elements, which can improve the expression level of recombination protein, and the rate of positive colonies in CHO cells. This effect may be related to the increase in transgene copy number, and the activity of recombinant protein is closely related to the level of mRNA containing MAR vector.

## CONFLICT OF INTEREST

The authors declare no competing financial interests.
